# Supplementary material for: The impact of internet usage preferences on labor income: Evidence from China
Source: PLoS One. 2024 Aug 6;19(8):e0308287. doi: 10.1371/journal.pone.0308287 (PMC11302926; doi:10.1371/journal.pone.0308287)
Supplement: Attachment — Submitted filename: Response to Reviewers.docx [file pone.0308287.s001.docx]

## Response to Reviewers

**Reviewer #1: The proposed paper is devoted to the impcat of internet usage preferences on labor income. The material is focused on Chinese region and Chinese economy specifics. The processed paper is well designed and existing knowledgap is specified through the relevant literature overview. The objectives of the paper are also relevant and the proposed methodology is suitable and well explained. The results part of the paper is rational and individual results and findings are relevant and supported by relevant arguments. The final discussion part of the paper is not well processed - it should be separted from the conclussion and key findings related to already published outputs should be highlighted. The final conclussion is too brief. There is no relevant information related to paper's limitations. Also some recommendations for future research are missing.**

**Author's Response:**Thank you very much for your recognition of my article and your valuable modifications. I have the following modifications in response to your modifications, please criticise and correct me.

1. Revision of the discussion part of the paper: In the context of the popularisation of Internet technology, the formation mechanism of Internet usage preference is discussed based on the technology acceptance model and the theory of biased technological progress, and a logical relationship diagram is drawn to make it easier for readers to understand the mechanism.

2. Modification of the conclusion of the thesis: (1) The limitation of data and the limitation of variable selection are added. (2) Pointed out the future research direction and suggestions from two aspects.

**Reviewer #2: This topic is in accordance with the context of 4.0 industrial revolution. The technology (Internet usage) has a significant contribution to the society and labor's income. Author(s) apply regression model (1) to estimate the internet usage on labor's income.
In terms of statistics and regression, this approach seems to be reasonable. However, the model does not clearly explain the mechanism of impact of technology (or internet use) on improving workers' income. Note that income must be linked to real economy activities. In equation 1, there is almost no mention of real economic factors. In other words, we need a model that explains the relationship between technology (the Internet) and income through the workings of the real economy.
Without theoretical background, the research resembles an econometrics exercise.**

**Author's Response:**

Thank you very much for your valuable comments on my article. In response to your comments, I have made the following changes, and I would be grateful for your criticisms and corrections.

1. Income is not linked to the real economy: the survey question "Do you use the Internet at your workplace to improve your work efficiency?" was found in the CGSS2017 database and its data was used as a measure of an individual's Internet use at work. survey question and used its data as a measure of an individual's internet use at work, Personal Internet Use at Work (PIUW). The variable showed statistical significance from the test of ZINB and CMP endogeneity model.

2. Modification of the influence mechanism of the thesis: In the discussion part of the thesis, the formation mechanism of Internet usage preference is discussed on the basis of the technology acceptance model and the theory of biased technological progress, and a logical relationship diagram is drawn to make it easier for readers to understand the mechanism.

3. Modification of the conclusion of the thesis: (1) The limitation of data and the limitation of variable selection are added. (2) Pointed out the future research direction and suggestions from two aspects.

**Reviewer #3: Using the data of China General Social Survey (CGSS) 2017, this paper uses Stata statistical software to build a model to analyze the impact of individual Internet use preference on labor income. It provides new evidence for understanding how Internet usage preferences affect labor income and highlights the importance of digital literacy, providing useful insights for policymakers and residents alike. I think the paper needs further revision.**

**Author's Response:**

Thank you very much for your valuable comments on my article. In response to your comments, I have made the following changes, and I would be grateful for your criticisms and corrections.

1. The paper mentions the importance of digital literacy, and can further explain the specific content of digital literacy to help readers better understand it. In addition, the purpose, motivation and innovation of this paper should be further emphasized.
2. In Section 3, please explain the model and functional equations in detail so that the reader can better understand the research methods.
3. For functional equation (1), please explain the meaning of "" in the equation.
4. Improve the clarity of the bar chart in Figure 1.
5. Literature should be updated to add the latest research results of famous journals in the past three years.

**Reviewer #4: The identification strategy needs to be revised to disentangle the effect of the internet on education and access to measure its effects on wages. The paper study an interesting topic but needs a better empirical strategy.**

**Author's Response:**

Thank you very much for your valuable comments on my article. In response to your comments, I have made the following changes, and I would be grateful for your criticisms and corrections.

This paper uses data on Internet use preferences multiplied with Educational attainment and Frequency of study in leisure time to derive the interaction term Improve Digital literacy (IDL) for all three. Through ZINB regression and CMP regression, the results show that enhancing the education of digital literacy and frequency of self-study related to Internet technology has a significant contributing effect on enhancing IDL (Table 8).

In addition, the influence of education level on the labour income effect of individual Internet use preference was clarified by building a mediation effect model.
