# Supplementary material for: The impact of internet usage preferences on labor income: Evidence from China
Source: PLoS One. 2024 Aug 6;19(8):e0308287. doi: 10.1371/journal.pone.0308287 (PMC11302926; doi:10.1371/journal.pone.0308287)
Supplement: Attachment — Submitted filename: renamed_87d8e.docx [file pone.0308287.s002.docx]

**Author's Response to Reviewer 3#**

Dear Reviewer 3#，Hello! I have made the following improvements to the article in response to your proposed changes:

(1) A description of the solution to Eq. (2) and the formula derivation process have been added in Section 3.3. The bayes:zinb model was also used for parameterization and comparative analysis.

(2) A covariate, PIUW, which is prone to zero inflation, was added to the model in order to enhance the convergence of the regression analysis.

(3) Subscripts were added to beta in equation (2).

(4) Added the analysis and interpretation of CGSS2017 data sources and their types in Section 3.1, and added information on the range of values and units of the variables in the descriptive statistics table of the variables in Section 3.2.

(5) The purpose and content of the empirical analysis were added in section 4

(6) Redrawn Figure 1 based on the percentage of data from INC.

(7) Graphs were added to sections 4.3 and 4.4 to facilitate comparisons by the reader.

Thank you again for your valuable comments! These comments have refreshed the article!

**Author's Response to Reviewer 4#**

Dear Reviewer 3#，Hello! I have made the following improvements to the article in response to your proposed changes:

(1) Added analysis and explanation of CGSS2017 data sources and their types in section 3.1;

(2) Information on the range of values and units of the variables was added to the descriptive statistics table of the variables in section 3.2.

(3) In section 3.3 the variables were analyzed for correlation using Spearman and Pearson methods.

(8) A description of the solution to Equation (2) and the formula derivation process was added in Section 3.3. The bayes:zinb model was also used for parameterization and comparative analysis.

(9) In section 4.5, “regional cable length” is chosen as a new instrumental variable, which passes the correlation and exogeneity tests; regression analyses are carried out with the help of the cmp model and the instrumental variables, and finally the marginal effects of IUF, OSN, OE, AOI and OB on the improvement of individual labor income are explained respectively.

The conclusions of the article are solidified by your valuable comments! Thanks again for your hard work!
