# Supplementary material for: The impact of internet usage preferences on labor income: Evidence from China
Source: PLoS One. 2024 Aug 6;19(8):e0308287. doi: 10.1371/journal.pone.0308287 (PMC11302926; doi:10.1371/journal.pone.0308287)
Supplement: Attachment — Submitted filename: renamed_44e3a.docx [file pone.0308287.s004.docx]

**Author's Response to Reviewer 3#**

Dear Reviewer 3#，Hello! I have made the following improvements to the article in response to your proposed changes:

1. The layout of formulas (3) and (5) has been re-edited.

2. There is no blank page after the adjustment of formula (5).

3. The first and third lines of the three-line table have been bolded.

4. The labels of the horizontal and vertical axes of the charts have been completed.

5. The graph has been redrawn to solve the problem of inconsistent thickness of horizontal and vertical coordinates.

Thank you again for your efforts!

**Author's Response to Reviewer 4#**

Dear Reviewer 4#，Hello! I have made the following improvements to the article in response to your proposed changes:

Figure 1 was plotted and explanatory text added to the article to explain the status (in per cent) of survey respondents' Internet usage preferences.

Thank you again for your efforts!
